# Evolutionary history and transmission dynamics of dengue virus type 2 in Africa

**DOI:** 10.3389/fmicb.2026.1782819

**Published:** 2026-04-30

**Authors:** Muhammad Bashir Bello, Safia S. Aljedani, Nafi’u Lawal, Abdulrahman Alasiri, Mustapha Umar Imam

**Affiliations:** 1 Department of Infectious Disease Research, King Abdullah International Medical Research Center, King Saud bin Abdulaziz University for Health Sciences, Ministry of National Guard Health Affairs, Riyadh, Saudi Arabia; 2 Center for Advanced Medical Research and Training, Usmanu Danfodiyo University, Sokoto, Nigeria; 3 Department of Artificial Intelligence and Bioinformatics, King Abdullah International Medical Research Center, King Saud bin Abdulaziz University for Health Sciences, Ministry of National Guard Health Affairs, Riyadh, Saudi Arabia; 4 Center for Vaccine Research and Biotechnology, Federal University of Lafia, Lafia, Nigeria

**Keywords:** Africa, dengue virus type 2, genetic diversity, genomic surveillance, phylogeography

## Abstract

**Introduction:**

Dengue virus serotype 2 (DENV-2) is a major driver of severe dengue outbreaks globally, yet its evolutionary history and transmission dynamics across Africa remain incompletely understood.

**Methods:**

To clarify its origins, introduction pathways, and patterns of regional spread, we compiled all available full-length DENV-2 genomes up to 16 July 2025, prioritizing African sequences while retaining representative global references. After quality filtering and down-sampling, a final dataset of 414 genomes was analyzed. Sequences were screened for recombination using RDP4 and analyzed with maximum-likelihood phylogenetics in IQ-TREE with 1,000 ultrafast bootstrap replicates. Temporal dynamics were inferred using time-scaled phylogenies reconstructed in BEAST under a strict molecular clock and exponential-growth coalescent prior, while viral dispersal was examined through discrete-trait phylogeographic analyses with Bayesian stochastic search variable selection. In parallel, single-nucleotide polymorphisms in the prM, E, and NS1 genes were characterized, and significant sites were mapped onto corresponding protein structures.

**Results and Discussion:**

Between 1964 and 2024, African DENV-2 diversity was shaped primarily by repeated introductions of the Cosmopolitan genotype, giving rise to geographically structured lineages. Lineage IIB, likely originating from La Réunion in the late 1970s, subsequently disseminated across West, Central, and East Africa, with Burkina Faso emerging as a key regional hub. Lineage IIA, also linked to La Réunion, diverged into two sub-lineages that expanded through East and Central Africa and extended into South Asia. In contrast, lineages IID and IIF were detected only sporadically, with limited geographic representation and no evidence of sustained regional expansion. Despite substantial genetic variation across prM, E, and NS1, relatively few significant non-synonymous mutations were identified, and critical N-glycosylation motifs remained conserved. This pattern suggests that epidemiological connectivity and repeated viral introductions, rather than sustained positive selection at major functional loci, are the dominant forces shaping DENV-2 spread in Africa. Overall, our findings highlight the central role of recurrent introductions and regional transmission networks in structuring DENV-2 circulation across the continent and emphasize the need for strengthened genomic surveillance to track lineage emergence and improve evidence-based dengue prevention and control strategies.

## Introduction

Dengue virus (DENV), a member of the Flaviviridae family, is a mosquito-borne pathogen responsible for one of the most widespread and rapidly escalating arboviral diseases globally (Zeng et al., 2021). The virus exists as four antigenically distinct but closely related serotypes (DENV-1 to DENV-4), each capable of causing a broad spectrum of clinical manifestations–from asymptomatic infection and classical dengue fever to severe outcomes such as dengue hemorrhagic fever and dengue shock syndrome (Harapan et al., 2020). Among these, DENV-2 is particularly notorious for its association with severe disease and frequent involvement in major outbreaks (Narvaez et al., 2025). It is genetically diverse, comprising six recognized genotypes (I-VI), each with unique geographical and evolutionary characteristics (Messina et al., 2019).

Traditionally, dengue has been endemic to Southeast Asia, the Western Pacific, and the Americas, where it has caused recurrent outbreaks and imposed a substantial public health burden (Harapan et al., 2019; Jagtap et al., 2023; Fernando et al., 2024; Taylor-Salmon et al., 2024). However, in recent decades, the virus has undergone notable geographic expansion, increasingly establishing local transmission in regions previously considered at low risk (Shepard et al., 2016). Among these emerging regions, Africa has historically been perceived as peripheral to the global dengue landscape due to limited reporting and presumed low disease burden. Yet, growing evidence now indicates a steady increase in dengue incidence across the continent, with DENV-2 identified as the predominant serotype implicated in several recent outbreaks spanning East, West, and Central Africa (Eltom et al., 2021). This shifting epidemiological trend demands the urgent need for a deeper understanding of DENV dynamics in Africa, particularly in the context of surveillance, outbreak preparedness, and vaccine strategy development.

Despite growing evidence of dengue transmission across Africa (Emeribe et al., 2021; Mwanyika et al., 2021), the evolutionary history and transmission patterns of the virus–particularly DENV-2–remain poorly understood. Several factors have contributed to this knowledge gap, including limited access to molecular diagnostic tools, weak surveillance systems, underreporting of cases, and the diagnostic overlap of dengue with other endemic febrile illnesses such as malaria, chikungunya, and yellow fever (Gainor et al., 2022; Lim et al., 2025). Recent outbreaks in East, West, and Central Africa have revealed the silent but sustained presence of DENV in urban and peri-urban areas, with studies suggesting the circulation of multiple viral lineages within and between countries (Alfsnes et al., 2021; Onoja et al., 2024). DENV-2, in particular, has demonstrated notable genetic diversity in Africa, with both the Cosmopolitan and Sylvatic genotypes documented in various regions (Nyathi et al., 2024), raising concerns about increased risks of severe disease, antibody-dependent enhancement, and complexities in deploying effective vaccines.

In this study, we analyze publicly available DENV-2 sequences from Africa to trace their evolutionary history, map transmission routes, and examine their connections to global lineages. These insights are essential for improving surveillance, guiding vaccine strategies, and strengthening outbreak preparedness across the continent.

## Materials and methods

### Sequence datasets, recombination analysis and maximum likelihood phylogeny

All available complete genome sequences of DENV-2 from African countries were retrieved from NCBI Virus database (Brister et al., 2015). Duplicate sequences were identified and removed. Only sequences with high coverage were included in the analysis, while those lacking sampling date information were excluded. Additional available DENV-2 sequences were obtained from dengue-endemic regions in Southeast Asia and South Asia. To ensure global representation, sequences from other regions were also included based on genotype diversity, geographic origin, and date of sample collection. Sylvatic DENV-2 strains were identified and excluded from the dataset to focus the analysis on human-endemic lineages. The datasets were subsequently down-sampled to minimize redundancy while preserving temporal, geographic, and genotypic diversity. The temporal range of the sequences spanned from 1964 to 2024. All selected sequences were aligned using MAFFT v7 (Katoh and Standley, 2013) with default parameters.

Recombination analysis was performed using RDP4 version 4.101 (Martin et al., 2015) to screen the aligned dataset prior to phylogenetic and phylodynamic analyses. Multiple detection methods implemented in RDP4 (including RDP, GENECONV, BootScan, MaxChi, Chimera, SiScan, and 3Seq) were applied under default settings. Recombination events supported by at least two methods with associated *p*-values below the recommended threshold (*p* < 0.05) were considered credible. Sequences identified as recombinant were excluded from further analysis to avoid potential bias in phylogenetic reconstruction, molecular clock estimation, and phylogeographic inference. Only non-recombinant sequences were retained for maximum likelihood phylogenetic reconstruction and subsequent Bayesian evolutionary analyses.

An initial maximum likelihood phylogenetic tree was constructed using IQ-TREE version 2.3.4 (Minh et al., 2020) under the GTR + F + I + R4 substitution model, selected based on model testing, with branch support assessed using 1,000 ultrafast bootstrap replicates. The resulting tree was used to evaluate overall phylogenetic structure, identify potential outliers, and confirm the genetic relationships among sequences. The temporal signal of the dataset was subsequently assessed using TempEst version 1.5.3 (Rambaut et al., 2016) by examining the correlation between root-to-tip genetic divergence and sampling time, to determine the suitability of the data for molecular clock analyses. Sequences exhibiting anomalous divergence patterns, if any, were carefully evaluated and excluded where appropriate. The final curated dataset comprised 414 non-recombinant sequences from 41 countries, spanning the period 1964–2024 (Supplementary Table S1).

### Bayesian reconstruction of DENV-2 evolutionary history

Bayesian phylogenetic inference was performed in BEAST version 1.10.4 (Suchard et al., 2018) to reconstruct the time-scaled evolutionary history of DENV-2 in Africa across 1964–2024. A strict molecular clock, the GTR + F + I + R4 substitution model, and a coalescent exponential growth prior were employed to model sequence evolution and demographic dynamics. The use of a strict molecular clock was supported by the temporal signal observed in the dataset, as assessed using TempEst, which showed a strong correlation between genetic divergence and sampling time. The relatively narrow range of estimated substitution rates further suggested limited rate heterogeneity among lineages, supporting the use of a strict clock over a relaxed molecular clock model. Two independent Markov Chain Monte Carlo (MCMC) runs of 50 million iterations each were conducted with BEAGLE acceleration, sampling every 50,000 steps. LogCombiner version 1.10.4 was used to merge runs post 10% burn-in, and convergence was assessed in Tracer version 1.7.2 (Rambaut et al., 2018) ensuring ESS > 200 for all parameters. A maximum clade credibility (MCC) tree was generated in TreeAnnotator version 1.10.4 and visualized with FigTree version 1.4.4.^1^

### Tracing the geographic dispersal of DENV-2

Discrete trait phylogeographic analysis was conducted in BEAST version 1.10.4 by assigning each DENV-2 sequence a country or region to model geographic spread. An asymmetric substitution model was applied to estimate directional transition rates between locations, while Bayesian Stochastic Search Variable Selection (BSSVS) was used to identify a parsimonious subset of significantly supported migration pathways. Bayes Factor (BF) analysis quantified the statistical support for each transition, with BF ≥ 3 interpreted as strong evidence for non-random dispersal.

### SNP calling and annotation

We selected prM, E, and NS1 for gene-level analysis due to their complementary structural, antigenic, and immune-modulatory roles in DENV-2 biology. While phylogenetic and phylodynamic analyses were performed using full-length genomes, SNP profiling was conducted separately for each gene. Nucleotide frequencies were calculated at each site relative to the reference sequence, followed by codon reconstruction to classify variants as synonymous or non-synonymous. Variants were considered epidemiologically significant if they reached a prevalence ≥ 10% and were observed in ≥2 countries and ≥2 years. Significant non-synonymous sites were subsequently mapped onto available protein structures to assess their spatial distribution.

We analyzed the same multiple sequence alignment used for the phylogeographic analysis, restricted to African sequences (non-African sequences removed; recombinants excluded as described above). A Singapore DENV-2 Cosmopolitan strain (KX380828.1) was used as the reference. We focused on three coding regions: prM (nt 283–780; 166 aa), E (nt 781–2265; 495 aa), and NS1 (nt 2266–3321; 352 aa). At each ungapped reference position, nucleotides were tallied across sequences, treating A/C/G/T as informative and excluding gaps or ambiguous bases on a per-site basis. For each alternate base, we recorded the alternate count (k), the informative total (n), the prevalence (*p* = k/n), and 95% Wilson confidence intervals. Using the gene reading frame, reference and alternate codons were reconstructed, translated, and classified as synonymous or non-synonymous. For each variant, nucleotide and amino acid changes, as well as codon position, were recorded. Countries and years associated with each variant were extracted from FASTA headers.

## Results

### Diversity of DENV-2 in Africa

**FIGURE 1:**
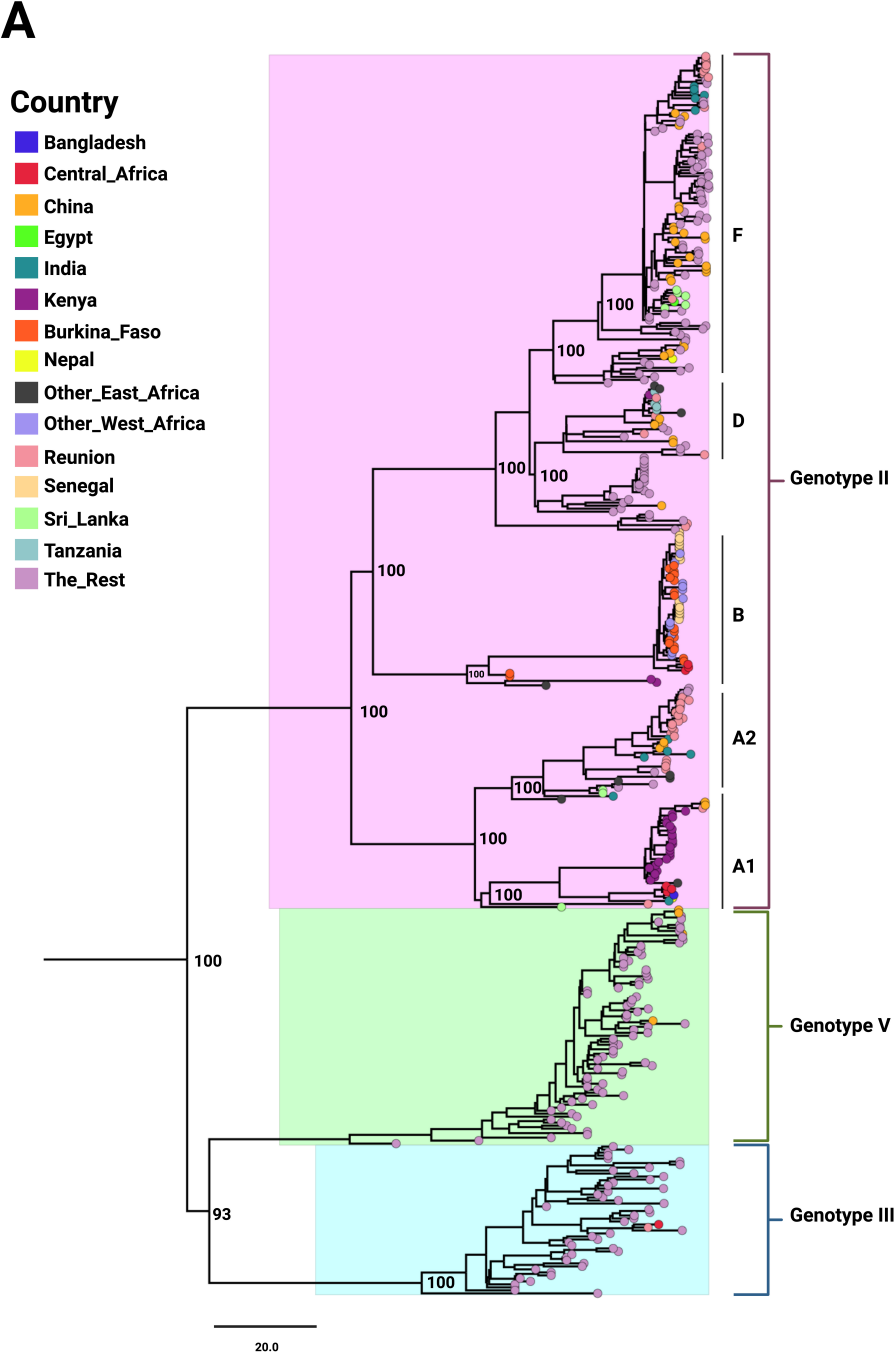
Maximum-likelihood phylogenetic tree of African DENV-2 sequences with global references. Tip colors indicate countries of origin, and major genotypes and lineages are annotated. Bootstrap values are shown at key nodes to highlight the robustness of inferred relationships. The tree is drawn to scale.

A total of 414 complete DENV-2 genome sequences from 41 African countries (Supplementary Table S1) were analyzed using maximum likelihood phylogenetic reconstruction. Root-to-tip regression analysis using TempEst demonstrated a strong temporal signal (Supplementary Figure S1), supporting the suitability of the dataset for molecular clock–based analyses. The sequences showed substantial genetic heterogeneity. Genotyping with the Genome Detective Tool identified the majority of African DENV-2 sequences as belonging to the Cosmopolitan genotype (Genotype II), which was subdivided into lineages A, B, D, and F (Figure 1). Within this genotype, lineages displayed distinct clustering patterns and variable branch lengths. A single Central African isolate was classified as Genotype III and clustered with a reference strain from La Réunion. Clear geographic structuring was observed: sequences from West Africa were assigned predominantly to Lineage B, with additional representation in East and Central Africa, while Lineage A comprised mainly East African sequences with some from Central Africa. Older isolates were positioned at the base of clades, whereas more recent isolates formed terminal clusters.

### Emergence and expansion of Lineage IIA

**FIGURE 2:**
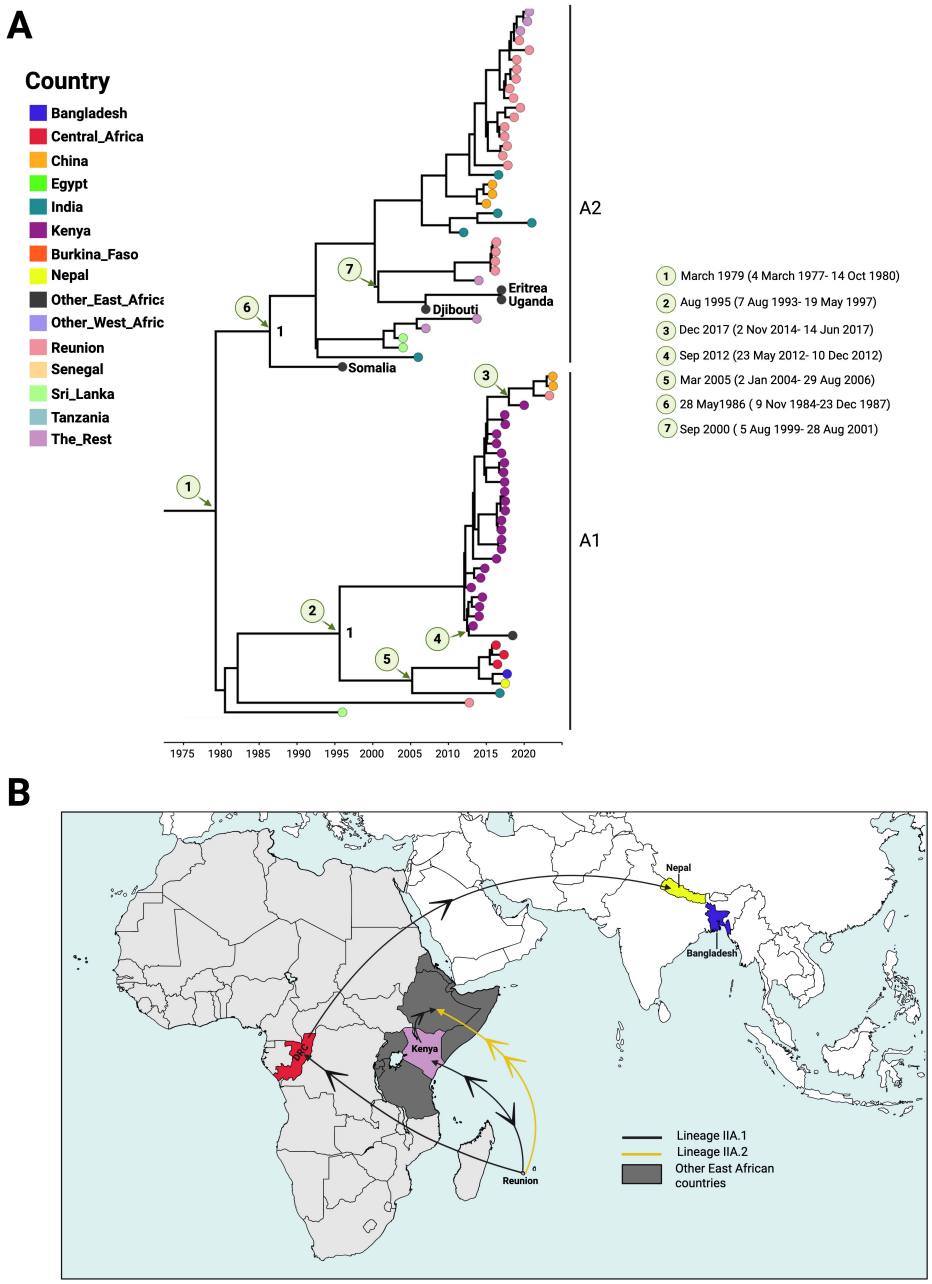
Origin and spread of DENV-2 Lineage IIA in Africa. **(A)** Time-scaled phylogeny of African DENV-2 Lineage IIA sequences. Estimated times to the most recent common ancestors (tMRCAs) for major African clusters are indicated, with posterior probabilities shown at key nodes. Sampling countries are distinguished by color coding at the tips. **(B)** Spatial dispersal patterns of DENV-2 Lineage IIA within Africa and beyond. Arrows indicate the inferred directions of viral movement between regions based on the phylogeographic reconstruction.

Within the DENV-2 Cosmopolitan genotype (Genotype II), African sequences assigned to Lineage IIA shared a common ancestor dated to approximately March 1979 (95% HPD: March 4, 1977 – October 14, 1980), with La Réunion identified as the most probable source (country probability = 0.53). These estimates are consistent with the overall root age of African DENV-2 at 1923 (95% HPD: 1919–1927), an evolutionary rate of 7.75 × 10^–4^ substitutions/site/year, and an exponential growth rate of 3.49 × 10^–3^ (Table 1). Phylogenetic reconstruction indicated diversification into two distinct sub-lineages, A1 and A2 (Figure 2).

**TABLE 1:** Evolutionary characteristics of DENV-2 in Africa.

Parameter	Median value	95% HPD	Effective sampling size
Age (root)	1923.45	1919.36–1927.25	493.5
Exponential population size	141.44	116.81–170.45	1483.5
Exponential growth rate	3.49 × 10^–3^	−6.80 × 10^–3^–1.29 × 10^–2^	5653.2
Evolutionary rate	7.75 × 10^–4^	7.45 × 10^–4^–8.08 × 10^–4^	276.8

Sub-lineage A1 included sequences linked to the 2017 dengue outbreak in Kenya. Bayesian analysis estimated its introduction into Kenya between August 7, 1993, and May 19, 1997 (95% HPD), with La Réunion identified as the most likely source (posterior probability = 1; country probability = 0.79). Evidence of transmission from Kenya back to La Réunion was also detected, with the TMRCA dated to mid-2017. This lineage was later detected in China in 2023. Sequences from Kenya subsequently spread to other East African countries, with a TMRCA around mid-2012. In addition, sequences from Central Africa (DRC, 2016–2017) appear to have been introduced as early as 2005 (95% HPD: January 1, 2004 – August 28, 2006), most likely from La Réunion (country probability = 0.65). These sequences shared a common ancestor dated to 2014 with South Asian sequences from Nepal and Bangladesh (posterior probability = 1).

Sub-lineage A2 was inferred to have been introduced into East Africa by May 28, 1986 (95% HPD: November 9, 1984 – December 23, 1987), again from La Réunion (posterior probability = 1; country probability = 1). A subsequent introduction occurred around late 2000 (95% HPD: August 5, 1999 – August 28, 2001), also traced back to La Réunion.

### Emergence and regional spread of Lineage IIB

**FIGURE 3:**
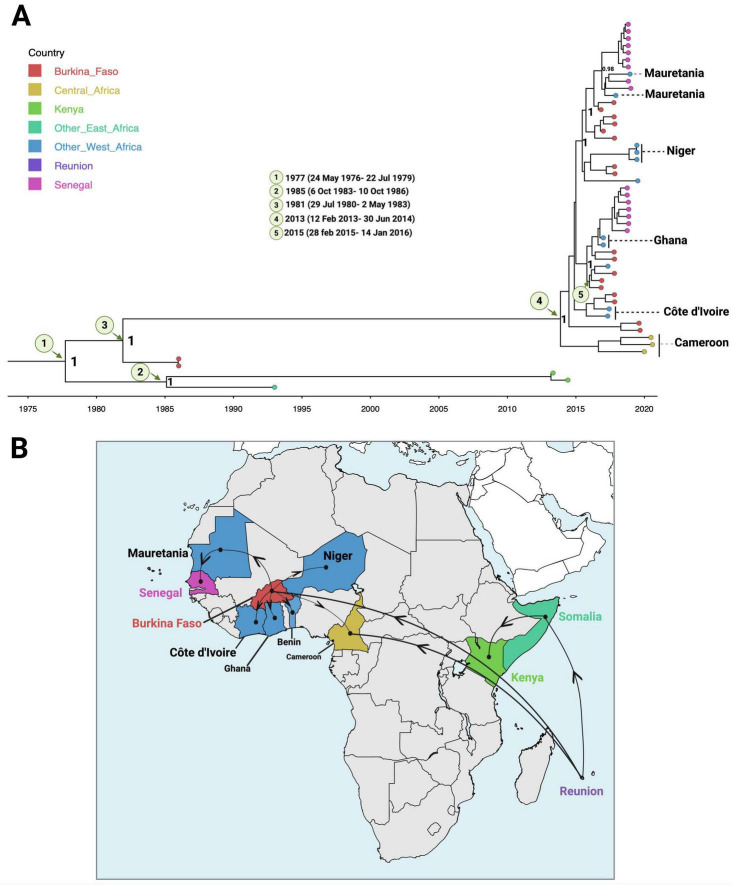
Phylogeography of DENV-2 Lineage IIB in Africa. **(A)** Bayesian time-scaled phylogenetic tree of DENV-2 Lineage IIB sequences, showing estimated times to the most recent common ancestors (tMRCAs). Sampling countries are represented by distinct tip colors, and posterior probability values are shown at key nodes. **(B)** Inferred origin and spatial spread of DENV-2 Lineage IIB across Africa. Arrows indicate the direction of viral movement between regions based on the phylogeographic reconstruction.

Lineage IIB of the Cosmopolitan genotype likely entered the African continent no later than September 27, 1977 (95% HPD: May 24, 1976 – July 22, 1979), with La Réunion implicated as the most probable source (posterior probability = 1; country probability = 0.59). From this initial introduction, the lineage spread into both East and West Africa (Figure 3).

In East Africa, the lineage was already circulating by 1985 (95% HPD: October 6, 1983 – October 10, 1986), although it was not formally reported until 1993. It was subsequently associated with dengue outbreaks in Kenya during 2013–2014. In West Africa, the earliest evidence of local transmission was traced to Burkina Faso by late 1981 (95% HPD: July 29, 1980 – May 2, 1983), preceding its first detection in 1986. Burkina Faso later emerged as a major hub for onward dissemination, with the TMRCA shared with Central African (Cameroonian) sequences dated to approximately October 13, 2013 (95% HPD: February 12, 2013 – June 30, 2014). Evidence of viral exportation from Burkina Faso to neighboring countries (including Ghana, Niger, Benin, Mauritania, and Côte d’Ivoire) was observed, with the earliest inferred TMRCA around 2015 (95% HPD: February 28, 2015 – January 14, 2016).

Phylogeographic reconstruction further supported multiple independent introductions into Senegal, including one from Ghana between June and December 2016 (posterior probability = 0.6; country probability = 1) and another from Mauritania in late 2016 (posterior probability = 1; country probability = 0.88).

### Limited detection of Lineages IID and IIF

**FIGURE 4:**
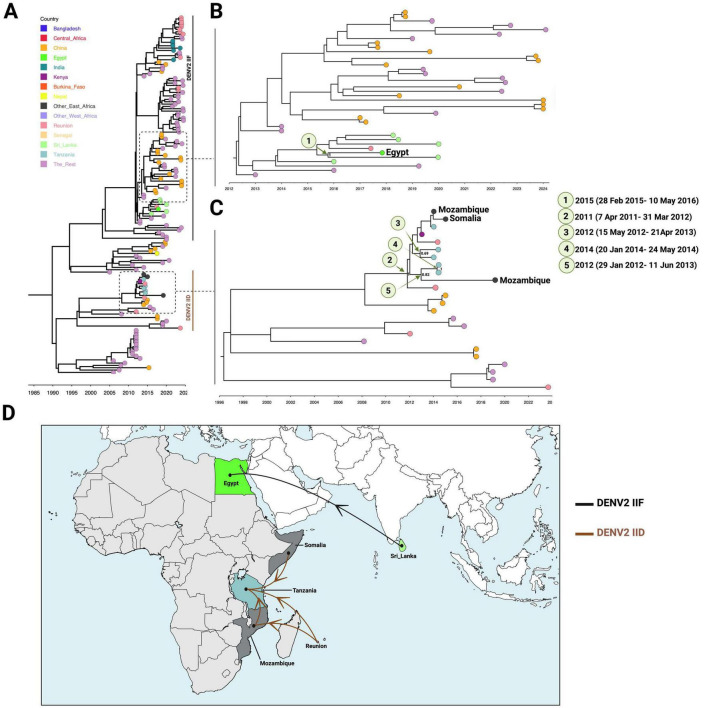
Evolutionary dynamics of DENV-2 Lineage IID and Lineage IIF in Africa. **(A)** Bayesian estimates of the tMRCA for African DENV-2 Lineage IID and Lineage IIF strains, highlighting the temporal origins of each lineage. **(B)** Expanded view of lineage IIF phylogeography. **(C)** Expanded view of the lineage IID phylogeography. **(D)** Inferred introduction events of Lineages IID and IIF into Africa, showing their emergence and early spread patterns across the continent.

Dengue virus serotype 2 Lineage IID was introduced into East Africa no later than September 7, 2011 (95% HPD: April 7, 2011 – March 31, 2012), with La Réunion as the most probable source (posterior probability = 1; country probability = 0.69). Multiple introductions into Tanzania were inferred during late 2012 and early 2014 (Figure 4). The lineage was also detected in Kenya in 2013. One isolate from this lineage, estimated to have diverged around late 2012 (95% HPD: January 29, 2012 – June 11, 2013), was identified in Mozambique in 2019.

Lineage IIF has only been recorded once in Africa, represented by an isolate from Egypt in 2017. This isolate was dated to an introduction between February 28, 2015 and May 10, 2016 (95% HPD), with a mean TMRCA of September 7, 2015. The most probable source was Sri Lanka (posterior probability = 0.99; country probability = 0.96).

### Mutation landscape in prM, E, and NS1

Across prM, E, and NS1, we identified 783 polymorphic sites, of which 326 (Supplementary Table S2) met the predefined significance criteria (prevalence ≥ 10% in ≥2 countries and ≥2 years). Among these, the median prevalence was 38.8% (range: 10.0%–99.2%). All significant variants met the epidemiological threshold, and none created or disrupted N-glycan sequons at E-67/E-153 or NS1-130/207.

In prM (nt 283–780; 166 aa), 118 polymorphic sites were detected, with 51 classified as significant–primarily synonymous (47/51), with 4 non-synonymous. The most prevalent were prM-153 (synonymous, L) at 98.5% (95% CI: 94.6–99.6) across 17 countries over 16 years, prM-63 (synonymous, D) at 90.8% (95% CI: 84.7–94.7) across 15 countries over 12 years, and prM-148 (synonymous, Y) at 83.2% (95% CI: 75.9–88.6) across 15 countries over 13 years.

**FIGURE 5:**
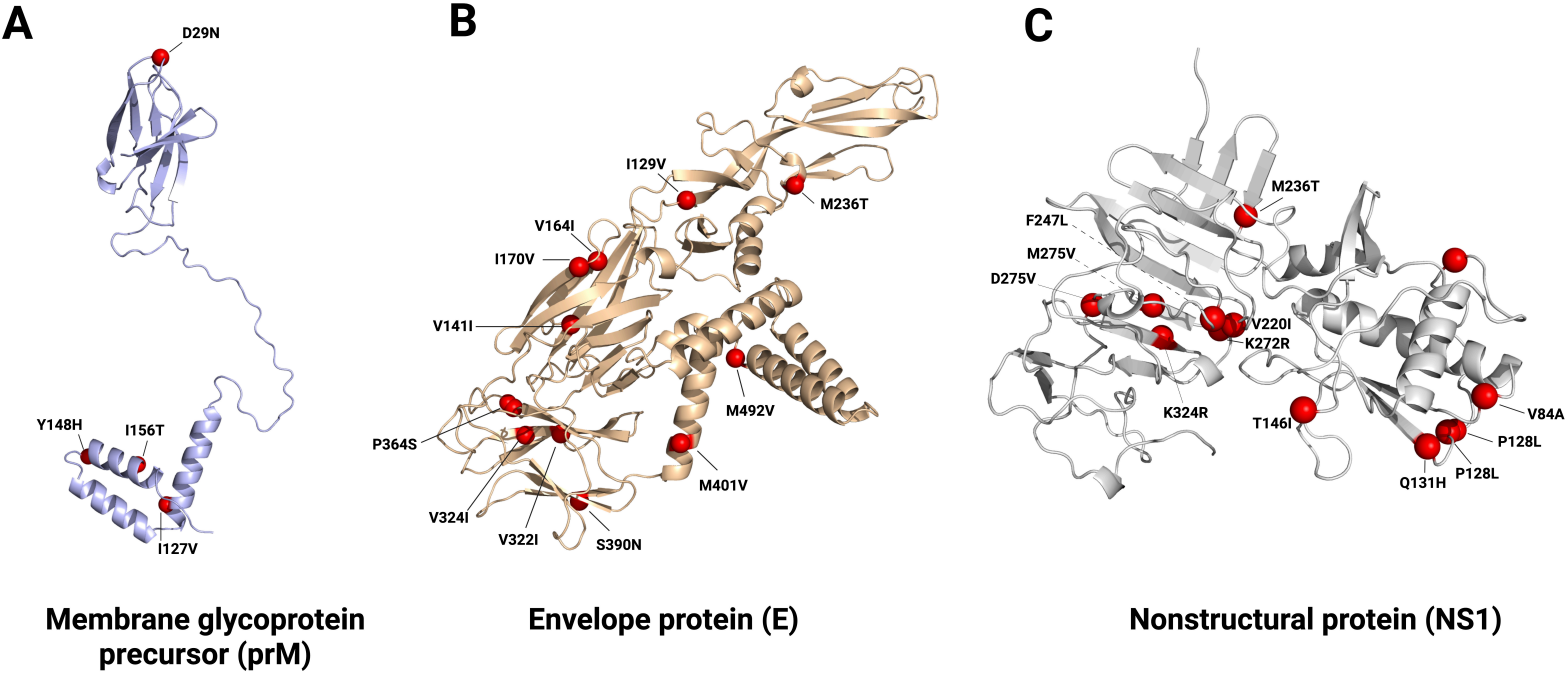
Three-dimensional structures of the **(A)** PrM, **(B)** E, and **(C)** NS1 proteins highlighting significant non-synonymous mutations in African DENV-2. Structural models display the location of amino-acid substitutions identified in African DENV-2 sequences, with key mutations mapped onto functional domains of PrM, E, and NS1 to illustrate their potential structural and phenotypic relevance.

In E (nt 781–2265; 495 aa), 380 polymorphic sites were identified, 167 of which were significant–predominantly synonymous (153/167), with 14 non-synonymous (Figure 5). The most frequent was E-236 (M → T, non-synonymous), detected at 99.2% (95% CI: 95.8–99.9) across 17 countries over 16 years, followed by E-310 (synonymous, K) and E-428 (synonymous, V), each at 99.2% (95% CI: 95.8–99.9) across 17 countries over 16 years. No significant variants altered the canonical E-67 or E-153 sequons.

In NS1 (nt 2266–3321; 352 aa), 285 polymorphic sites were identified, of which 108 were significant–largely synonymous, with 12 non-synonymous. The most prevalent were NS1-75 (synonymous, L) and NS1-179 (synonymous, C), each at 99.2% (95% CI: 95.8–99.9) across 17 countries over 16 years, and NS1-182 (synonymous, K) at 98.5% (95% CI: 94.6–99.6) across 17 countries over 16 years.

Overall, significant variants were geographically and temporally widespread, with a median distribution across 8 countries (range: 2–17) and 6 years (range: 2–16). Significant non-synonymous mutations identified in prM, E, and NS1 were further mapped onto their respective protein structures to highlight their spatial distribution (Figure 5).

## Discussion

Despite the rising incidence and geographic expansion of dengue across Africa (Gainor et al., 2022), genomic data from the continent remain strikingly sparse. As of 16th July 2025, only 1,304 complete DENV genomes from Africa were available in the GenBank–fewer than 5% of the 31,558 genomes globally. Many countries in northern, central, and southern Africa have not contributed a single full genome, and more than half of available sequences originate from La Réunion (Supplementary Figures S2, S3). This paucity of data severely limits the ability to track viral introductions, monitor evolutionary changes, and understand transmission dynamics within and across African regions. In this study, we reconstructed the evolutionary history and transmission patterns of DENV-2, which accounted for 43% of all African sequences. Our analyses reveal new insights into lineage diversity and cross-border viral movement while underscoring the urgent need to expand routine genomic surveillance to strengthen outbreak preparedness and vaccine implementation.

Our large-scale reconstruction shows that Africa is not merely a recipient of external viral introductions but also a contributor to onward dissemination. Phylogenetic and phylogeographic analyses reveal repeated introductions, regional diversification, and sustained local transmission that together define a heterogeneous and dynamic landscape. These findings broaden the global dengue narrative–traditionally dominated by Asia and Latin America–and highlight Africa’s increasingly important role in DENV-2 evolution and global spread.

The predominance of the Cosmopolitan genotype (Genotype II) mirrors global patterns, where its evolutionary success has been attributed to high viral fitness, vector compatibility, and efficient human mobility networks (Yenamandra et al., 2021; Amorim et al., 2023; Gräf et al., 2023). Within Africa, this genotype diversified into at least four distinct lineages (A, B, D, F), many of which established endemic or episodic transmission. Phylogenetic interspersion of African and Asian/Indian Ocean strains indicates multiple independent introductions, consistent with reports from Asia and South America (Drummond et al., 2006; Ashall et al., 2023).

Geographic structuring further defines these lineages: Lineage B dominates in West Africa, while Lineage A is largely restricted to East Africa. Similar spatial segregation has been observed for other arboviruses such as Zika and Chikungunya (Volk et al., 2010; Faria et al., 2017), suggesting shared ecological and epidemiological drivers. In particular, the distribution and population dynamics of mosquito vectors, especially *Aedes aegypti* and *Aedes albopictus*, likely play a key role in shaping these patterns. While vector genomic data were not included in this study, integrating viral phylogeography with vector population structure and ecology would provide a more comprehensive understanding of arbovirus spread. Our data suggest that Lineage B was introduced from La Réunion into Burkina Faso no later than the early 1980s and subsequently spread through West and Central Africa, establishing Burkina Faso as a regional transmission hub likely facilitated by trade and mobility networks. This echoes findings for DENV-1, where Senegal and Burkina Faso were identified as epicenters of viral export (Dieng et al., 2021; Letizia et al., 2022). Multiple introductions of Lineage B into Senegal further demonstrate opportunities for repeated re-establishment, reflecting porous borders and limited vector control.

Cryptic transmission is a recurring theme, exemplified by Lineage IID in Mozambique, which circulated undetected for approximately 7 years after its introduction around 2012. Delays in detection are common due to under-reporting, misdiagnosis with malaria or other febrile illnesses, and limited molecular capacity (Amarasinghe et al., 2011; Baje et al., 2025). Similar silent spread has been documented in Southeast Asia (Tran et al., 2023) and Saudi Arabia (Bello et al., 2025). These findings emphasize the need to integrate genomic surveillance with routine febrile illness monitoring to uncover hidden transmission chains and to deploy adaptable vaccine technologies capable of mitigating outbreak potential (Bello et al., 2025).

La Réunion emerged as a recurrent source of introductions into Africa, consistent with its role as a regional arboviral hotspot (Mavian et al., 2018). Viral exchanges are likely facilitated by air travel, inter-island migration, and commerce, as seen in the Pacific (Li et al., 2010). Likewise, the identification of Lineage IIF in Egypt, closely related to Sri Lankan strains, highlights the risks of transcontinental movement through global travel and trade. While no onward spread has been observed, Egypt’s position as a major hub demands heightened border health surveillance.

From an evolutionary perspective, the relatively old TMRCA of Lineage IIB (1977) with deep branching suggests long-standing endemic persistence and slow genetic drift, whereas more recent TMRCAs for Lineages IID and IIF indicate emerging outbreak lineages. These dynamics resemble Latin American patterns, where older endemic strains coexist with newly introduced variants (Mir et al., 2014). However, we note that sparse and uneven sampling across much of Africa may introduce bias into phylogeographic reconstructions. Limited sequence availability from several regions can obscure intermediate transmission steps, potentially leading to overrepresentation of well-sampled locations as apparent sources of viral spread. As a result, inferred introduction routes and migration pathways should be interpreted with caution, as they may reflect sampling intensity rather than true transmission dynamics. In addition, incomplete temporal and geographic coverage may affect tMRCA estimates, potentially biasing lineage age estimates either upward or downward depending on the distribution of available sequences. These limitations highlight the need for more comprehensive and geographically representative genomic surveillance to improve the accuracy of phylogeographic and evolutionary inferences.

SNP analyses complement these phylogeographic insights by revealing broad coding conservation across prM, E, and NS1. Most significant variants (≥10% prevalence in ≥2 countries and ≥2 years) were synonymous, and canonical N-glycan motifs at E-67/E-153 and NS1-130/207 remained intact. These motifs are functionally critical for viral entry, assembly, and endothelial interactions (Kudlacek et al., 2021), and their preservation signifies strong structural constraints. Non-synonymous changes were distributed across E-protein domains rather than clustering at the fusion loop or glycan sites, suggesting their utility as lineage markers rather than indicators of adaptive remodeling (Huang et al., 2010; El-Kafrawy et al., 2016). In NS1, variation spared glycan motifs but included C-terminal substitutions known to modulate stability and secretion (Ogire et al., 2021). Together with classic evidence that few E sites, notably around E-390, show genotype-specific selection signals (Twiddy et al., 2002), these findings suggest that epidemiological factors (mobility, vector ecology, surveillance gaps) have primarily driven DENV-2 spread in Africa, rather than repeated selection on key functional loci.

In conclusion, this study provides one of the most comprehensive molecular epidemiological reconstruction of DENV-2 in Africa to date, revealing multiple introductions, structured transmission patterns, cryptic persistence, and cross-border viral movement. SNP profiling highlights conservation of key glycan motifs alongside lineage-specific markers useful for surveillance. Expanding genomic sampling–particularly from underrepresented countries–combined with standardized metadata, rapid data sharing, and functional assays of prevalent non-synonymous variants will be critical for uncovering hidden transmission, informing outbreak response, and ensuring that Africa contributes fully to global dengue preparedness and vaccine design.

## Data Availability

The original contributions presented in this study are included in the article/Supplementary material, further inquiries can be directed to the corresponding author.

## Footnotes

1
http://tree.bio.ed.ac.uk/software/figtree/

## Supplementary material

Supplementary Figure S1 Assessment of temporal signal using TempEst.: **(A)** Residual plot from the root-to-tip regression analysis showing the distribution of residuals across sequences. **(B)** Root-to-tip regression of genetic divergence against sampling time, demonstrating a positive correlation consistent with clock-like evolution. **(C)** Summary of regression parameters, including sampling date range, estimated slope, correlation coefficient (R^2^), and other model statistics.

Supplementary Figure S2 Number of global vs. African full dengue virus genomes available in the GenBank (As of 16th July 2025). **(A)** Distribution of the genomes by serotype. **(B)** Distribution of the genomes by African subregion.

Supplementary Figure S3 Map showing the total number of dengue virus type 2 genomes per African country identified from public databases. These counts include all available sequences prior to filtering (including sylvatic strains and recombinant sequences) and before downsampling for phylogeographic analysis. Countries with no available sequences are shown in green. The distribution highlights uneven sampling across the continent, with most sequences originating from a limited number of locations.
