# Supplementary figures and images for: Evolutionary history and transmission dynamics of dengue virus type 2 in Africa
Source: Front Microbiol. 2026 Apr 30;17:1782819. doi: 10.3389/fmicb.2026.1782819 (PMC13171821; doi:10.3389/fmicb.2026.1782819)

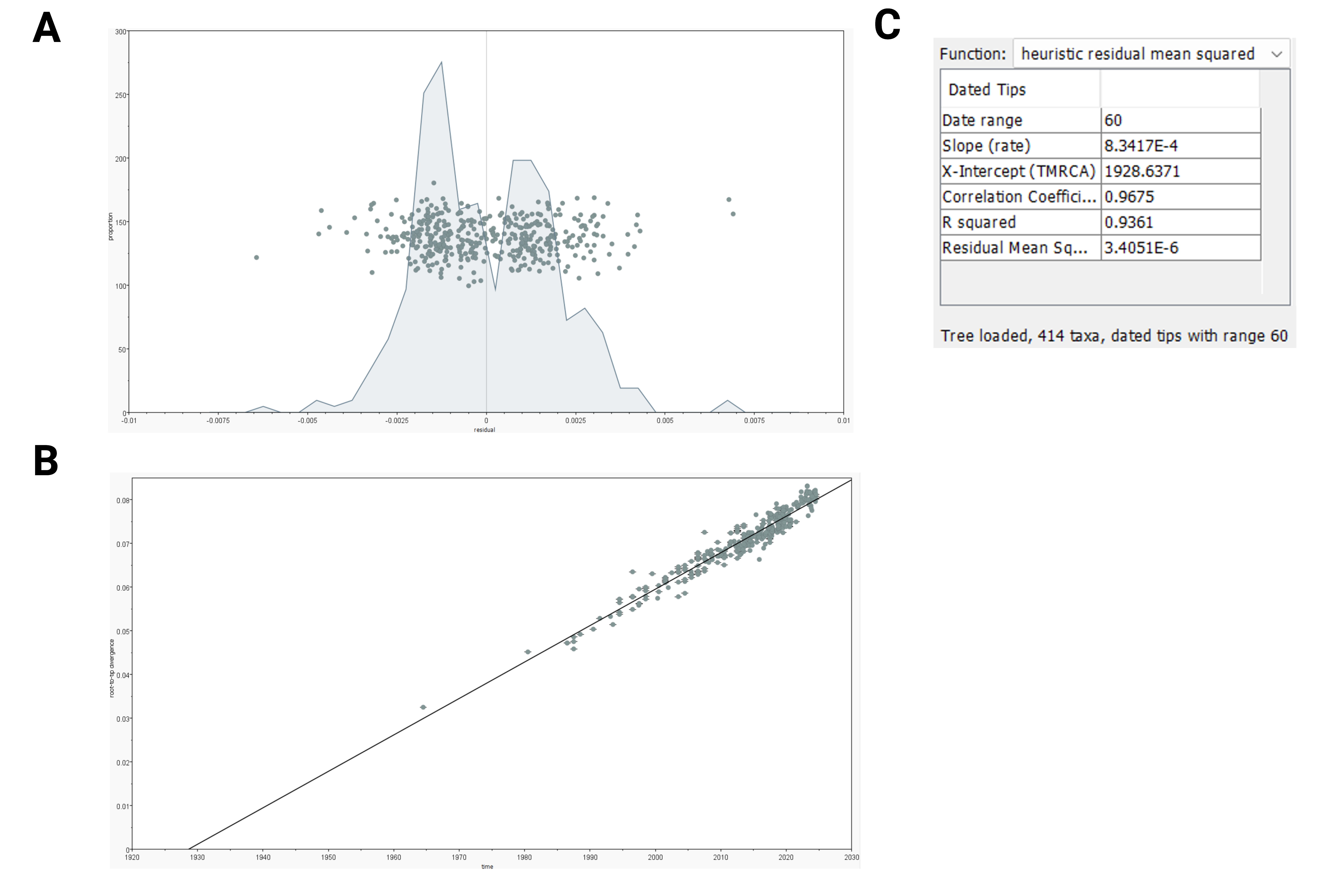

Supplement: Supplementary Figure S1 — Assessment of temporal signal using TempEst.: (A) Residual plot from the root-to-tip regression analysis showing the distribution of residuals across sequences. (B) Root-to-tip regression of genetic divergence against sampling time, demonstrating a positive correlation consistent with clock-like evolution. (C) Summary of regression parameters, including sampling date range, estimated slope, correlation coefficient (R2), and other model statistics. [file Image_1.jpeg]

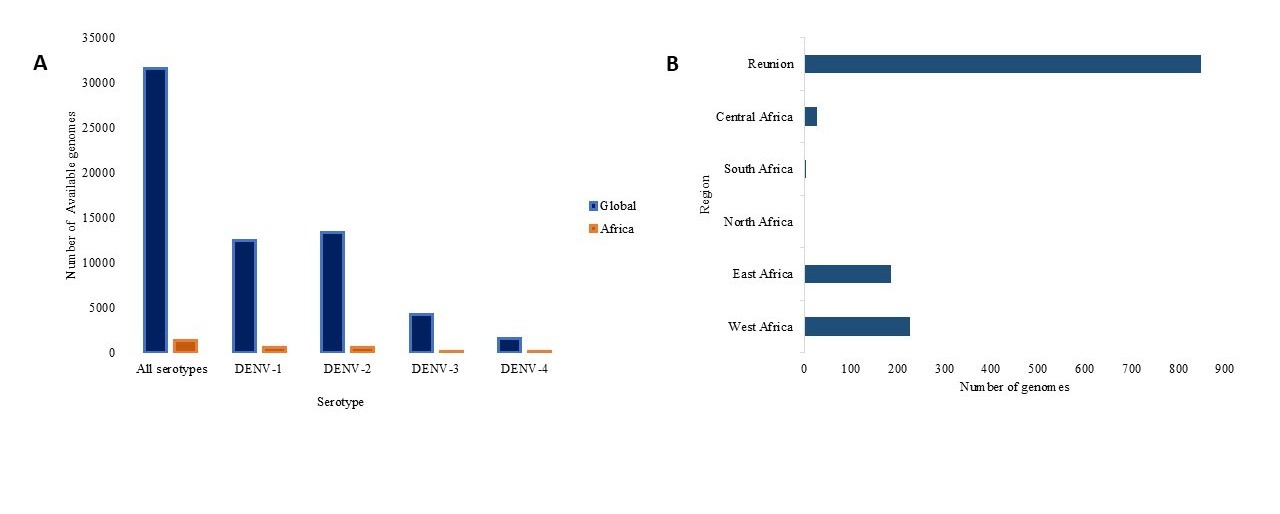

Supplement: Supplementary Figure S2 — Number of global vs. African full dengue virus genomes available in the GenBank (As of 16th July 2025). (A) Distribution of the genomes by serotype. (B) Distribution of the genomes by African subregion. [file Image_2.jpeg]

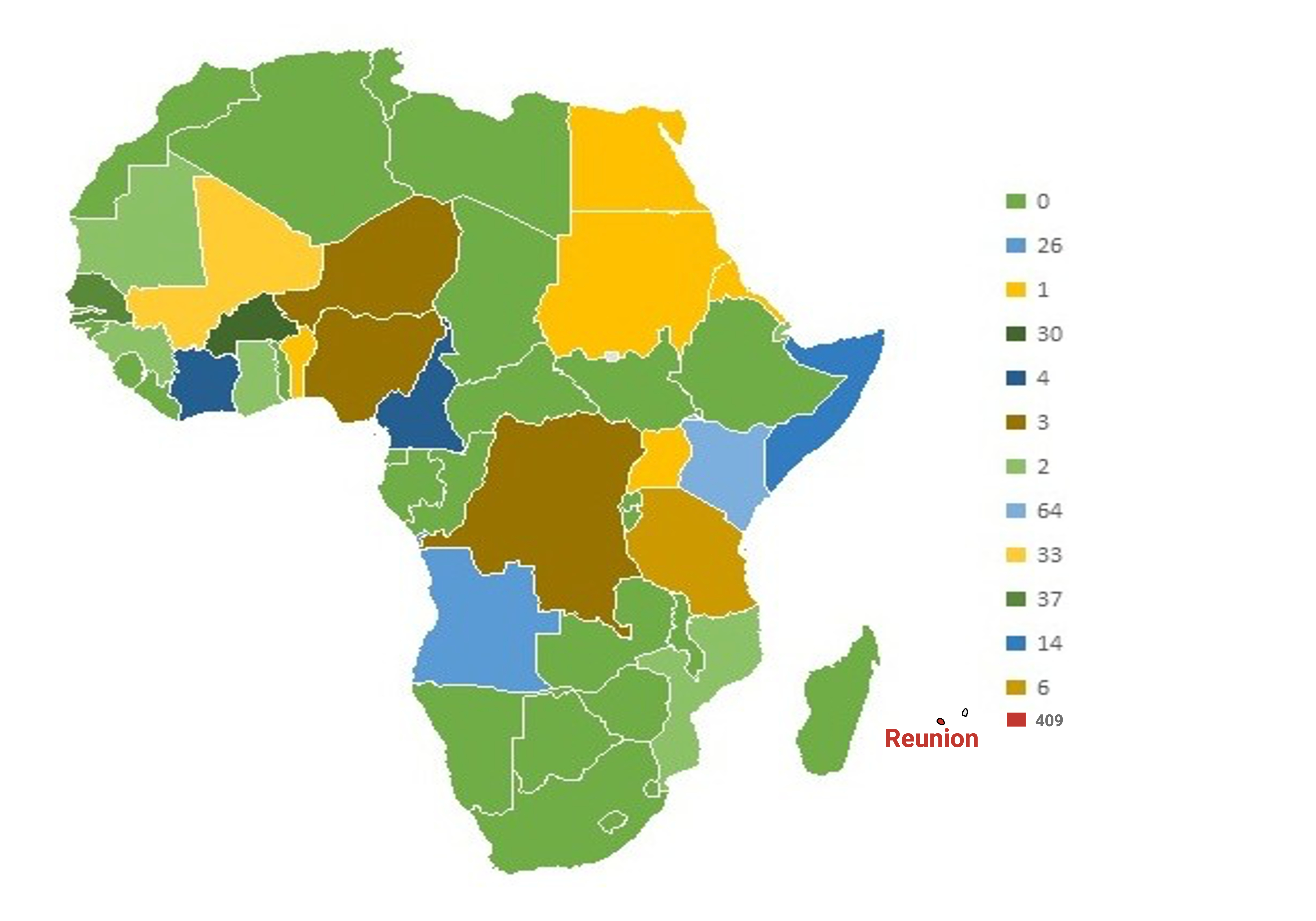

Supplement: Supplementary Figure S3 — Map showing the total number of dengue virus type 2 genomes per African country identified from public databases. These counts include all available sequences prior to filtering (including sylvatic strains and recombinant sequences) and before downsampling for phylogeographic analysis. Countries with no available sequences are shown in green. The distribution highlights uneven sampling across the continent, with most sequences originating from a limited number of locations. [file Image_3.jpeg]
